# Minimizing Hemorrhage Risk Strategies in Cervical Pregnancy—Stepwise Pharmacologic Priming and Delayed Surgical Evacuation: A Narrative Review

**DOI:** 10.3390/jcm14217489

**Published:** 2025-10-22

**Authors:** Victor Bogdan Buciu, Gabriel Florin Răzvan Mogoș, Nicolae Albulescu, Sebastian Ciurescu, Dorin Novacescu, Mihai Ionac, Abhinav Sharma, Nilima Rajpal Kundnani, Denis Serban

**Affiliations:** 1 Doctoral School, “Victor Babes” University of Medicine and Pharmacy Timisoara, E. Murgu Square, No. 2, 300041 Timisoara, Romania; victor.buciu@umft.ro (V.B.B.); sharma.abhinav@umft.ro (A.S.); 2 Department of Surgery, University of Medicine and Pharmacy of Craiova, 200349 Craiova, Romania; 3 Division of Internal Medicine II, “Victor Babes” University of Medicine and Pharmacy, E. Murgu Square, No. 2, 300041 Timisoara, Romania; nicolae.albulescu@umft.ro; 4 Centre for Molecular Research in Nephrology and Vascular Disease, “Pius Brânzeu” County Emergency Hospital, Department of Cardiology, 300041 Timisoara, Romania; 5 Department of Microscopic Morphology, Discipline of Histology, “Victor Babes” University of Medicine and Pharmacy Timisoara, E. Murgu Square, No. 2, 300041 Timisoara, Romania; novacescu.dorin@umft.ro; 6 Department of Scientific Research Methodology, “Victor Babes” University of Medicine and Pharmacy Timisoara, E. Murgu Square, No. 2, 300041 Timisoara, Romania; mihai.ionac@gmail.com; 7 University Clinic of Internal Medicine and Ambulatory Care, Prevention and Cardiovascular Recovery, Department VI-Cardiology, “Victor Babes” University of Medicine and Pharmacy, 300041 Timisoara, Romania; 8 Research Centre of Timisoara Institute of Cardiovascular Diseases, “Victor Babes” University of Medicine and Pharmacy, 300041 Timisoara, Romania; 9 Department of Obstetrics-Gynaecology, Discipline of Obstetrics-Gynecology, “Victor Babes” University of Medicine and Pharmacy Timisoara, E. Murgu Square, No. 2, 300041 Timisoara, Romania; denis.serban@umft.ro

**Keywords:** CP, heterotopic pregnancy, Doppler ultrasound, methotrexate, mifepristone, uterine artery embolization, hysteroscopy, suction curettage, fertility preservation

## Abstract

**Background**: CP (CP) and HCP (HCP) are rare and high-risk conditions, often historically managed with radical intervention and associated with hemorrhage and fertility loss. **Objective**: To summarize current evidence on the conservative, fertility-preserving management of cervical and heterotopic cervical pregnancies and to illustrate a stepwise pharmacologic protocol applied in our tertiary center. **Methods**: A narrative literature review (PubMed, Scopus, Web of Science; inception—July 2025) was conducted using the following key terms: “CP,” “HCP,” “methotrexate,” “mifepristone,” “misoprostol,” “uterine artery embolization,” “hysteroscopy,” and “Doppler ultrasound.” We integrated a personal institutional case that applied stepwise pharmacologic priming, Doppler-guided surveillance, and delayed evacuation. **Results**: Evidence—primarily from case reports and small series—supports conservative, multi-modal strategies combining systemic or local methotrexate ± mifepristone, timed to Doppler-confirmed vascular regression, before surgical intervention. Adjuncts such as misoprostol, hysteroscopic resection, balloon tamponade, and uterine artery embolization further reduce hemorrhage risk while maintaining fertility. Our case utilized a novel, incremental dosing strategy of mifepristone followed by methotrexate, a week-long interval to confirm vascular involution via Doppler, and delayed suction curettage with minimal blood loss. **Conclusions**: Conservative, imaging-guided management is promising for reducing hemorrhagic complications and preserving fertility in CP/HCP. Future multicenter registries and standardized Doppler-based protocols are urgently needed to refine decision-making and optimize outcomes.

## 1. Introduction

Cervical pregnancy (CP) is a rare and potentially life-threatening subset of ectopic gestation, estimated to represent less than 1% of all ectopic pregnancies, with reported incidence ranging from approximately 1:1000 to 1:18,000 pregnancies [1,2]. Heterotopic CP (HCP) remains exceptionally rare, with less than 150 cases having ever been reported worldwide to date. A bibliometric overview indicates a steady increase in publications addressing CP and HCP over the past two decades, reflecting growing clinical recognition and a shift toward conservative, fertility-preserving management approaches. When existing alongside a concurrent intrauterine gestation, the condition becomes an HCP, an exceedingly rare clinical entity for which standardized diagnostic and therapeutic guidelines are lacking [3,4].

Advances in transvaginal ultrasound have enabled earlier detection of CP—even before the onset of hemorrhagic complications—yet management remains a formidable challenge. The dual preservation of maternal safety and reproductive potential demands a highly individualized, often multimodal approach. Historically, therapeutic strategies span from systemic or local administration of methotrexate (MTX) to invasive options like curettage, uterine artery embolization, or even hysterectomy in cases of uncontrolled bleeding [1,5].

The combined use of methotrexate and mifepristone as a conservative treatment for CP has been described in limited case series. For instance, García et al. reported the successful management of cervical (and interstitial) ectopic pregnancies using intramuscular methotrexate (50 mg/m^2^) and oral mifepristone (600 mg), with some cases additionally managed by minimally invasive methods such as uterine artery embolization and dilation and curettage [6]. Similarly, observational studies have shown that combining oral mifepristone and systemic misoprostol with methotrexate can result in the effective resolution of CP when β-hCG levels are comparatively low (<5000 mIU/mL) [1,7].

While systemic methotrexate is often the backbone of conservative therapy, the literature also describes local administration strategies—such as ultrasound-guided intra-amniotic injections of MTX with or without potassium chloride—to expedite trophoblastic demise and reduce vascular risks [8].

Although several case reports and small series exist, no consensus guidelines have been established for CP [1,2,6]. A narrative review of available evidence is warranted to contextualize our case and highlight pharmacologic and surgical strategies that minimize hemorrhage while preserving fertility.

### 1.1. Aim of Study

The purpose of this narrative review is to summarize current evidence on the conservative, fertility-preserving management of CP and HCP pregnancies as primary and secondary objectives, respectively. Furthermore, the manuscript aims to illustrate a stepwise pharmacologic protocol applied in our tertiary center and suggest a practical framework based on our experience and the current literature.

### 1.2. Ethics and Consent

All personal identifiers from the collected data were removed to ensure anonymity, and patient confidentiality was maintained in accordance with the General Data Protection Regulation (GDPR, EU 2016/679). The study adhered to the ethical principles of the Declaration of Helsinki (2013 revision). This study was approved by the Ethics Committee of Victor Babes University of Medicine and Pharmacy under registry number 4/2 September 2025.

## 2. Methods

This paper follows the Scale for the Assessment of Narrative Review Articles (SANRA) guidelines and provides a narrative synthesis of the published literature. It is not a systematic review or meta-analysis, but a descriptive narrative review supplemented by a representative case from our tertiary center to illustrate the clinical application of conservative management strategies. A comprehensive electronic search of PubMed/MEDLINE, Scopus, and Web of Science was performed from database inception to July 2025. The following keywords and Boolean operators were applied: “CP” OR “cervical ectopic pregnancy” OR “heterotopic pregnancy” OR “HCP” AND (“methotrexate” OR “mifepristone” OR “misoprostol” OR “uterine artery embolization” OR “surgical management” OR “hysteroscopy” OR “Doppler ultrasound”). The literature search covered the period from January 2000 to July 2025, ensuring the inclusion of all contemporary diagnostic and therapeutic reports.

The search was restricted to English-language articles published in peer-reviewed ISI-indexed journals. Eligible publication types included case reports, case series, retrospective and prospective studies, and reviews. Conference abstracts, non-peer-reviewed sources, and reports without sufficient clinical detail were excluded.

Given the descriptive nature of this review and the heterogeneity of the available evidence (primarily case reports and small series), a formal PRISMA flowchart and risk-of-bias assessment were not applicable. Instead, findings were grouped thematically into pharmacological approaches, interventional and surgical strategies, imaging guidance, and reproductive outcomes. To complement the literature review, we integrated a case from our tertiary center that illustrates a stepwise conservative strategy.

Furthermore, based on the current understanding of the literature and our prior experience, we propose a management framework to be followed. This was elaborated over the course of 9 years and a total of 47 cases of CP treated, and based on available medical and interventional infrastructure in our tertiary center.

### 2.1. Quality of Evidence Appraisal

The quality of available evidence on cervical and HCP remains intrinsically limited. Most published data consist of isolated case reports and small, retrospective series, often with heterogeneous inclusion criteria, treatment regimens, and outcome definitions. Prospective protocols and randomized trials are absent. Consequently, much of the literature provides anecdotal evidence rather than generalizable conclusions.

Several limitations should be emphasized. First, publication bias is substantial, with successful conservative cases more likely to be reported than failures or severe complications, leading to an overestimation of efficacy and the underreporting of risks. Second, most studies provide short-term outcomes (resolution of pregnancy, avoidance of hysterectomy) but lack systematic reporting of long-term reproductive results, particularly live birth rates after conservative therapy. Third, dosing regimens for pharmacologic agents such as methotrexate and mifepristone vary widely across studies, with no standardized protocols, making cross-study comparison difficult. Fourth, outcome measures are inconsistently defined—some studies use hemorrhage volume, others highlight the need for transfusion or hysterectomy, and others only describe “conservative success,” preventing meta-analysis or pooled conclusions.

The level of evidence is therefore low (Level IV–V, according to the Oxford Centre for Evidence-Based Medicine criteria), derived almost entirely from observational studies and expert opinions. Despite encouraging reports of uterine preservation and subsequent conception, the true safety and effectiveness of different regimens remain uncertain.

### 2.2. General Considerations of Results

CP is a rare form of ectopic implantation, accounting for less than 1% of all ectopic gestations, with even fewer instances coexisting with an intrauterine pregnancy—a condition known as HCP [4,9]. The dual imperatives—prevent catastrophic hemorrhage and address patient reproductive goals—make management highly individualized.

### 2.3. Diagnosis and Imaging Modalities

The early and accurate diagnosis of CP pregnancy is critical to reduce the risk of catastrophic hemorrhage and to enable fertility-preserving interventions. Transvaginal ultrasound (TVUS) remains the cornerstone of diagnosis, allowing the precise localization of the gestational sac within the cervical canal. Key sonographic features include an empty uterine cavity (or a coexisting intrauterine gestation for HCP), presence of a gestational sac below the level of the internal os, absence of the “sliding sign” (lack of mobility of the sac with gentle probe pressure), and increased peritrophoblastic vascularity on color Doppler imaging [10,11,12,13,14].

Doppler assessment is particularly valuable, as increased vascular flow surrounding the gestational sac correlates with hemorrhagic risk and may guide the timing of the intervention [13,14].

Magnetic resonance imaging (MRI) has been described as an adjunct in suspected cases of abnormal placentation or deep trophoblastic invasion, though its use is generally limited to complex or equivocal cases [15,16,17,18]. Hysteroscopy has also been reported as both a diagnostic and therapeutic tool, permitting direct visualization of the cervical sac and targeted resection under ultrasound guidance [19,20].

Taken together, TVUS with Doppler represent the most practical and reproducible diagnostic modality, with additional imaging reserved for selected cases. Our experience supports the role of serial Doppler not only for initial diagnosis but also as a dynamic tool to monitor treatment response and determine optimal timing for surgical intervention.

Early instrumentation of vascular cervical implantation is hazardous; conversely, once serial Doppler demonstrates clear peritrophoblastic flow regression, evacuation becomes predictably safer. Throughout this review, we therefore interpret therapies through the lens of timing by vascularity, local expertise, and hemodynamic stability.

### 2.4. Pharmacological Approaches

In order to better understand the therapeutic arsenal at hand, we devised an explanatory subsection of the current available treatment options for the termination of pregnancy, in this case, ectopic cervical. Pharmacologic therapy aims to induce trophoblastic regression, minimize vascularity, and preserve fertility. Three main agents are reported: mifepristone, systemic methotrexate (MTX), and locally administered MTX. Each has distinct pharmacodynamic mechanisms, clinical indications, and limitations [6,21,22,23,24].

### 2.5. Mifepristone

Mifepristone is a competitive progesterone receptor antagonist. By blocking progesterone action at the endometrial and decidual level, it induces decidual necrosis, detachment of the trophoblast, and sensitizes myometrium to prostaglandins [25,26,27,28]. In CP, its role is less well established than in medical abortion, but case reports and small series suggest it can be effective in combination with methotrexate or misoprostol [29,30]. Its main advantages are oral administration, predictable onset of action, and selective induction of regression without immediate instrumentation. Limitations include variable efficacy in advanced gestations and limited evidence for its use as monotherapy. Contraindications include chronic adrenal failure, long-term corticosteroid use, bleeding disorders, and anticoagulant therapy [2,6,30].

Comparative selection and expected speed of regression are discussed in the Comparative Insights subsection.

### 2.6. Systemic Methotrexate

Methotrexate, a folate antagonist inhibiting dihydrofolate reductase, blocks DNA synthesis in rapidly dividing trophoblastic cells. It is the most widely reported agent in ectopic pregnancy, typically administered intramuscularly (50 mg/m^2^ or 100 mg fixed dose) [21,31]. Its advantages include broad availability, well-studied efficacy, and the ability to induce regression without surgery. Disadvantages include systemic toxicity (hepatic, hematologic, and gastrointestinal), delayed resolution, and lower efficacy in cases with high β-hCG (>10,000 mIU/mL) or advanced gestation [32,33,34,35]. Contraindications are well established: breastfeeding, chronic liver or renal disease, blood dyscrasias, peptic ulcer disease, and immunodeficiency [21,35].

Context-dependent effectiveness and timing relative to definitive procedures are addressed in the Comparative Insights subsection.

### 2.7. Local Methotrexate

Local injection of methotrexate directly into the gestational sac under ultrasound or hysteroscopic guidance allows a targeted cytotoxic effect with reduced systemic exposure [7,22,36]. Often combined with potassium chloride (KCl) to arrest embryonic cardiac activity, local MTX has been reported to reduce vascularity more rapidly and minimize systemic side effects [8]. Advantages include lower systemic toxicity and higher efficacy for cervical gestations with strong vascular supply. Disadvantages include technical complexity, need for operator expertise, and limited availability of guided injection facilities. There is also a risk of incomplete effect, requiring additional systemic MTX or surgical evacuation [5,9,23].

Comparative advantages, including anticipated pace of devascularization and setting requirements, are detailed in the Comparative Insights subsection.

All aforementioned pharmacological considerations are summarized in Table 1.

The table reflects data synthesized from published case reports, case series, and reviews on cervical and heterotopic pregnancy management. The indications and contraindications are not exhaustive and should be interpreted in the context of patient-specific factors and local resource availability. Evidence predominantly consists of case reports and small series (Level IV–V, Oxford CEBM). No standardized dosing regimens exist across studies.

In summary, pharmacologic priming is used to create a safer vascular milieu for later evacuation; modality selection should be interpreted alongside Doppler-defined vascularity and center-specific expertise.

### 2.8. Surgical and Interventional Options for HCP

Before continuing, we emphasize again that any definitive procedures should be timed to the vascular milieu created by conservative priming; Doppler evidence of attenuated peritrophoblastic/cervical stromal flow guides when instrumentation can be performed with lower bleeding risk.

Suction curettage is effective after pharmacologic priming when Doppler confirms diminished peritrophoblastic flow. In this setting, intraoperative adjuncts (e.g., Foley balloon tamponade) provide immediate hemostasis and permit short inpatient or outpatient recovery, with low transfusion rates reported in early series [10,35].

Hysteroscopic resection enables direct visualization and targeted removal of the cervical gestational sac with concurrent coagulation of bleeding sites. Reports describe successful use after medical priming and with ultrasound guidance, with low intraoperative blood loss and short stays [19,38,39,40]. This option is particularly valuable in centers with advanced endoscopic expertise.

Uterine artery embolization offers pre-emptive or rescue hemostasis and integrates with delayed curettage when bleeding risk is high. Case series report effective control of hemorrhage with uterine preservation and resumption of menses; subsequent pregnancies have been documented in selected cohorts. Protocols that combine embolization with intra-arterial or systemic methotrexate have been described [41,42,43,44]. In selected high-risk patients, intra-arterial infusion of methotrexate during UAE has also been reported to accelerate trophoblastic regression.

Balloon tamponade (e.g., Foley catheter) immediately after evacuation secures hemostasis. In a series of 13 first-trimester cervical pregnancies managed with suction curettage plus balloon tamponade, rapid bleeding control and uterine preservation were reported [45].

Taken together, these interventional options illustrate a paradigm shift: whereas hysterectomy was once the default management for CP, the combination of medical priming and minimally invasive surgical or radiologic interventions now allows high rates of uterine preservation and favorable reproductive outcomes. Comparative selection among these options is presented in the Comparative Insights subsection.

### 2.9. Comparative Effectiveness of Available Strategies

Comparative insights from the literature highlight important distinctions between available strategies. Systemic methotrexate is the most pragmatic first-line option given its broad availability, though it acts more slowly and is less effective at high β-hCG levels [21,31,32]. Local methotrexate achieves faster and more targeted regression but is technically demanding and limited to specialized centers [7,22,35]. Curettage performed without prior regression carries prohibitive hemorrhagic risk, yet when delayed until vascular flow subsides, it can be performed safely [10,34,35]. Uterine artery embolization remains the most reliable hemostatic tool, though its use depends on access to interventional radiology [41,42,43,44,46,47]. Hysteroscopy allows precise resection with concurrent coagulation but is feasible only in centers with advanced endoscopic expertise [38,39,40].

### 2.10. Fertility Preservation and Reproductive Outcomes

One of the principal goals in managing CP and HCP is the preservation of future fertility. Historically, cervical pregnancies were frequently complicated by uncontrollable bleeding [48]. One series reported hysterectomy in nearly 40%, effectively removing any chance of future fertility [34]. However, improvements in early ultrasound detection and conservative treatments have transformed outcomes. For instance, a retrospective study of 11 cases treated with methotrexate plus mifepristone reported 100% success without the need for surgical intervention [49]. Moreover, a comprehensive review noted that dilation and curettage alone carried approximately a 40% risk of necessitating hysterectomy, whereas combined methotrexate-based approaches and adjunct techniques have significantly lowered such rates [4].

Published reports indicate that successful conservative treatment of cervical CP does not preclude future conception. For example, in one retrospective review, the success rate of methotrexate-based therapy reached 81.3%, with combined conservative procedures (e.g., MTX plus adjunctive methods) increasing effectiveness to up to 91%, all without the need for hysterectomy [50]. Fertility outcomes appear most favorable in patients treated with systemic or local methotrexate, particularly when combined with interventions like uterine artery embolization or timed curettage [51].

Literature findings support that stepwise pharmacological priming followed by delayed evacuation can achieve resolution while minimizing hemorrhage risk [49]. Although long-term reproductive follow-up is not yet available, the absence of intraoperative trauma or excessive bleeding provides an optimistic outlook for future fertility.

### 2.11. Summary of Current Level of Evidence

To better contextualize our findings, we reviewed published manuscripts addressing cervical and heterotopic cervical pregnancies. While most available evidence consists of isolated case reports, several series provide valuable insight into the effectiveness of conservative pharmacological regimens, minimally invasive surgical techniques, and adjunctive interventions such as uterine artery embolization. These reports highlight trends in uterine preservation, complication rates, and subsequent reproductive outcomes, underscoring the shift away from historically high hysterectomy rates toward fertility-sparing approaches. A summary of selected case series is presented in Table 2.

Despite encouraging outcomes, important knowledge gaps remain: there is no consensus on Doppler thresholds that define a safe window for curettage or hysteroscopy, methotrexate dosing regimens vary widely, and long-term fertility outcomes—particularly live birth rates—are incompletely reported. Addressing these gaps is essential for developing standardized, evidence-based management protocols.

## 3. Personal Experience

**Figure 1:**
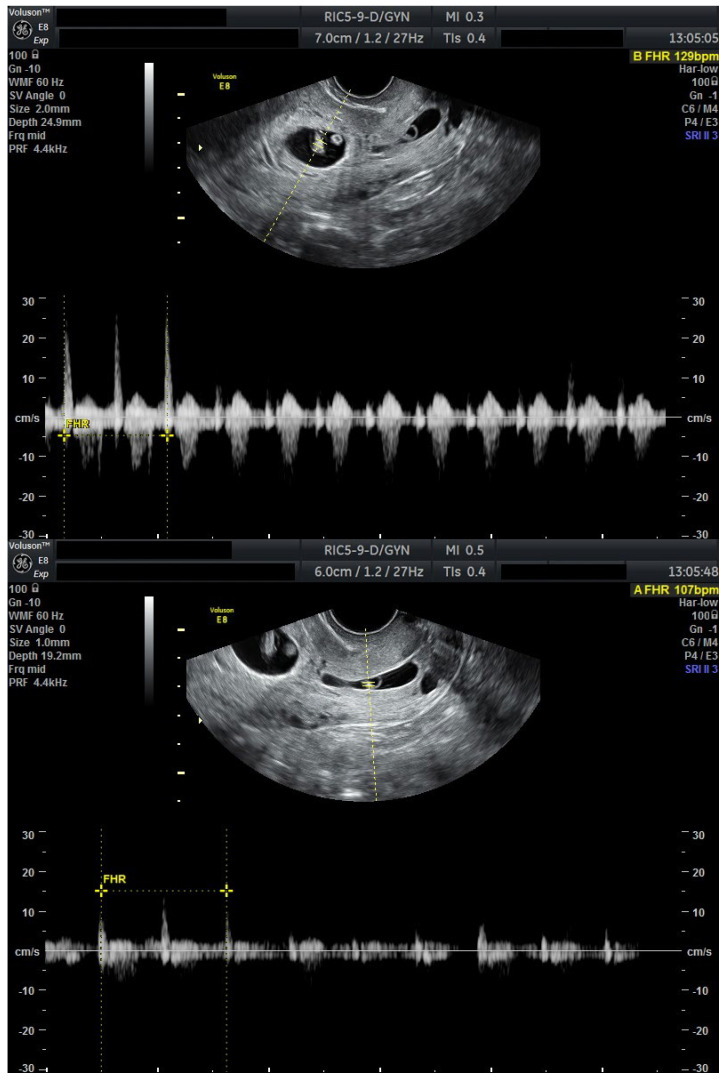
Ultrasound evaluation at diagnosis with viable heterotopic embryos (Day 0). Combined ultrasound of both gestational sacs. Transvaginal ultrasound evaluation: sagittal view through the uterus and cervical canal. FHR present in both embryos. Patient information is anonymized.

We present a personal case from our tertiary center in western Romania as an example of applicable findings from this review. A 32-year-old woman presented to the Municipal Clinical Emergency Hospital of Timișoara with first-trimester vaginal bleeding. Transvaginal ultrasound revealed a viable intrauterine gestation with a seven-week-old embryo with positive cardiac activity, as well as a simultaneous viable CP, confirming the diagnosis of HCP (Figure 1).

The patient first received mifepristone 200 mg orally at time of diagnosis (Day 0). At 48 h, repeat ultrasound demonstrated persistent cardiac activity in both the intrauterine and cervical gestations.

A second, higher dose of mifepristone 600 mg was then administered at this time (Day 2). Another 48 h later (Day 4), the CP no longer demonstrated cardiac activity, whereas the intrauterine pregnancy remained viable, but severely bradycardic (FHR = 67 bpm).

**Figure 2:**
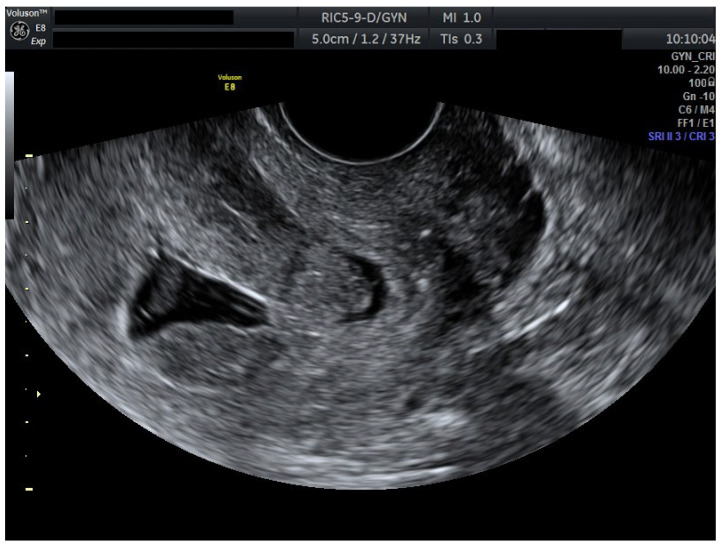

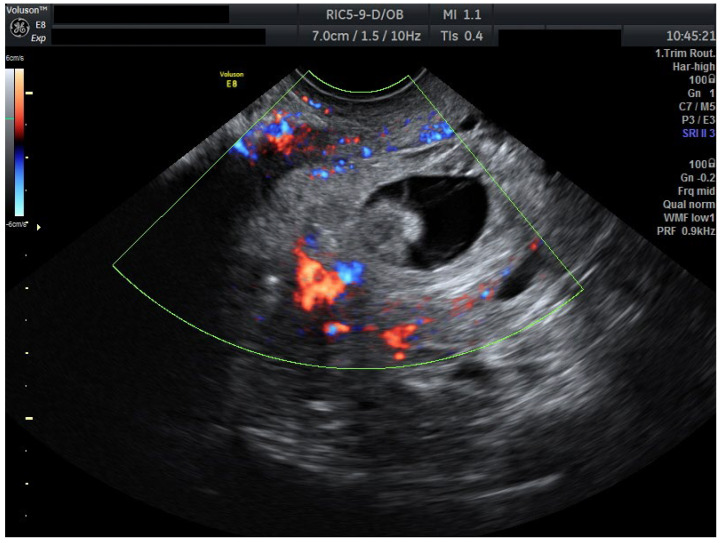
Ultrasound results after methotrexate 100 mg (Day 6). Transvaginal ultrasound evaluation: both sagittal view through the uterus and transverse view through the cervical canal. A total of 48 h after methotrexate 100 mg (Day 6): both embryos without cardiac activity. Cervical stroma with vascularity. Patient information is anonymized.

To ensure complete trophoblastic regression in both pregnancies, systemic methotrexate 100 mg was administered intramuscularly at this time (Day 4). A follow-up ultrasound at 48 h (Day 6) confirmed the absence of cardiac activity in both gestations, disorganized gestational sacs, consistent with successful termination. Cervical stromal vascularity identified on color Doppler was suggestive for trophoblastic remnants (Figure 2).

Serial ultrasound over 72 h documented progressive closure of feeding vessels and reduction in cervical stromal vascularity.

**Figure 3:**
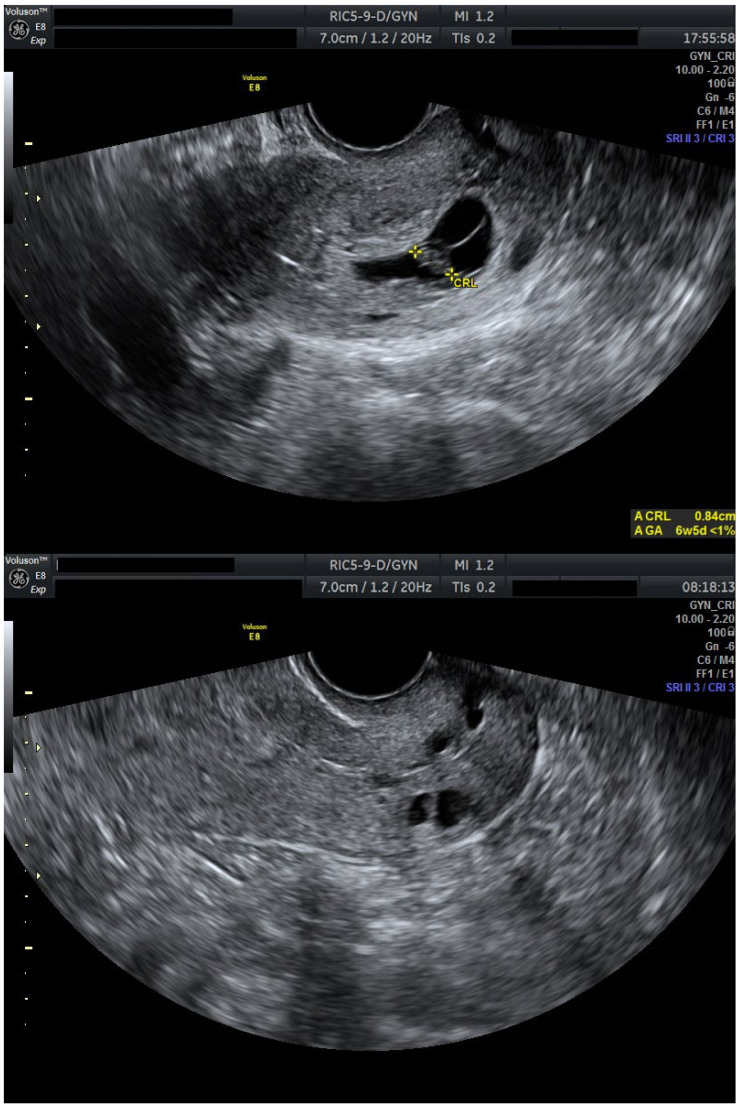
Cervical canal before and after suction curettage (Day 13). Transvaginal ultrasound evaluation: sagittal view through the cervical canal. A total of 24 h after initiation of misoprostol expulsion therapy. Impacted gestational sacs in the cervical canal before curettage and the empty cervical canal immediately after suction curettage. Patient information is anonymized.

After 72 h, the patient was re-evaluated. Cervical stromal vascularity presented no remaining gross vascularity. Subsequently, starting at the time of this ultrasound evaluation, the patient received misoprostol 0.2 mg intravaginally, every 6 h. This induced partial expulsion of the intrauterine pregnancy into the cervical canal, where it became impacted adjacent to the cervical gestational sac. Despite continued uterotonic administration, the cervical gestation did not expulse. Thus, a suction curettage was performed. The procedure was uneventful, with minimal blood loss, and post-procedure ultrasound confirmed complete evacuation of both gestations and restoration of a normal endometrial cavity and cervical canal (Figure 3).

**Figure 4:**
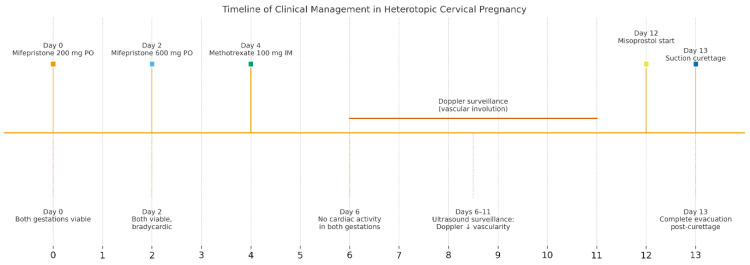
Timeline of clinical management in a case of HCP.

The patient’s recovery was uncomplicated under further uterotonic (misoprostol 0.2 mg every 8 h, intravaginally) to complete the expulsion of minimal residue and antibiotics (ceftriaxone f. 1 g once every 12 h, i.v.) (Figure 4). She was discharged in a stable condition, with β-hCG levels showing appropriate decline on dynamic follow-up.

Day numbering is relative to the first mifepristone dose (Day 0). Doses: mifepristone 200 mg PO (Day 0) and 600 mg PO (Day 2); methotrexate (MTX) 100 mg IM (Day 4); misoprostol initiation (Day 12); suction curettage (Day 13). “Doppler surveillance (Days 6–11)” denotes serial TVUS showing progressive reduction in peritrophoblastic/cervical stromal flow and absent embryonic cardiac activity prior to instrumentation. Abbreviations: MTX, methotrexate; TVUS, transvaginal ultrasound; FHR, fetal heart rate; and IUP, intrauterine pregnancy. Anti-D prophylaxis and hemostasis adjuncts (e.g., Foley balloon, uterotonics) were available per institutional protocol.

At three-month follow-up, the patient reported complete resumption of regular menstrual cycles. No complications or residual trophoblastic tissue were detected on ultrasound, and β-hCG levels had returned to non-pregnant values. At the time of manuscript submission, the patient had not yet attempted conception but expressed a desire for future pregnancy, and counseling regarding the optimal interpregnancy interval was provided.

### 3.1. Proposed Practice Framework: Sequence and Timing

Based on our current interpretation of the literature and prior experience, we propose the following framework of practice. Management must proceed in a short, ordered sequence, in order to avoid hemorrhage while preserving fertility. First, confirm diagnosis and quantify peritrophoblastic/cervical stromal flow by transvaginal ultrasound with color Doppler, documenting gestational age, cardiac activity, and baseline β-hCG. Second, initiate conservative priming—most commonly systemic methotrexate in stable patients, or local (intra-sac/intra-cervical) methotrexate where expertise allows; mifepristone can be used as first line if methotrexate is not available. Third, reassess on a predefined schedule (e.g., 48–72 h) with ultrasound/Doppler; when Doppler demonstrates clear attenuation of cervical/peritrophoblastic flow and embryonic cardiac activity is absent, proceed. Misoprostol can be used as uterotonic adjuncts per local protocol. Fourth, perform definitive evacuation—suction curettage or hysteroscopic resection—with immediate balloon tamponade available to secure hemostasis; when baseline bleeding risk is high or resources permit prophylaxis, integrate uterine artery embolization before or after evacuation. Fifth, follow β-hCG to resolution, administer anti-D immunoglobulin when indicated, and provide fertility counseling and contraception advice tailored to patient goals.

### 3.2. Limitations

This review is limited primarily by the quality of the available evidence. Most reports on CP and HCP consist of isolated case reports or small retrospective series, with marked heterogeneity in patient characteristics, treatment regimens, and outcome reporting. The absence of randomized or prospective comparative studies precludes firm conclusions regarding optimal pharmacologic protocols, the timing of interventions, or standardized Doppler thresholds for vascular regression. Fertility outcomes are inconsistently reported, often without long-term follow-up, which limits the ability to evaluate the true reproductive prognosis after conservative management. Our personal experience adds practical context but reflects a single case which cannot be generalized across different clinical settings. Moreover, local resource availability—such as lack of intra-amniotic methotrexate injection capability—may influence therapeutic decisions, further limiting external reproducibility.

### 3.3. Future Perspectives

Future research should aim to establish multicenter registries or collaborative databases dedicated to CP. Such efforts would allow the standardized collection of diagnostic criteria, treatment details, hemorrhagic outcomes, and fertility results, providing a more robust evidence base for management algorithms. Prospective studies should also explore the role of serial Doppler monitoring as a decision-making tool, ideally defining quantitative thresholds for safe surgical intervention. Comparative analyses between systemic versus local methotrexate, and between hysteroscopic, embolization-based, and curettage strategies, are needed to clarify relative efficacy and safety. Advances in imaging—including contrast-enhanced ultrasound and MRI—may further refine risk stratification. Ultimately, the integration of pharmacologic priming, imaging-based timing, and minimally invasive interventions into standardized, evidence-driven protocols has the potential to transform care, reducing hemorrhage risk while safeguarding fertility.

## 4. Conclusions

CP remains a rare but high-risk condition where hemorrhage and fertility loss are central concerns. Evidence from the literature, supported by our tertiary center’s experience, indicates that conservative, stepwise strategies can provide safe and effective outcomes. Pharmacologic priming with agents such as mifepristone and methotrexate, followed by short-term Doppler surveillance to document vascular involution, allows for delayed and safer surgical evacuation when required. Adjunctive methods including misoprostol, hysteroscopic techniques, and uterine artery embolization further expand the therapeutic arsenal. Although high-quality comparative data are lacking, the paradigm is shifting away from radical surgery toward individualized, fertility-preserving care. Future studies should aim to define reproducible Doppler ultrasound parameters that can objectively guide clinicians in determining the optimal and “safe” timing for surgical intervention following pharmacologic priming.

## Figures and Tables

**Table 1:** Comparative overview of pharmacological options for the management of cervical and heterotopic pregnancies.

Agent and Route	Mechanism of Action	Advantages	Disadvantages/Risks	Level of Evidence	Best Suited for	Contraindications
Mifepristone (oral)	Progesterone receptor antagonist → decidual necrosis, increased prostaglandin sensitivity	Oral, predictable onset, effective adjunct to MTX, avoids immediate instrumentation	Limited data in CP/HCP; variable effect in advanced GA; not validated as monotherapy	Case reports, small series	Early CP/HCP with desire to delay intervention	Adrenal insufficiency, chronic steroid use, bleeding disorders, anticoagulant therapy
Methotrexate (systemic)	Folate antagonist, inhibits DNA synthesis in trophoblast	Widely available, well-studied in ectopic pregnancy, non-invasive	Systemic toxicity (hepatic, renal, hematologic, GI); delayed resolution; reduced efficacy with β-hCG > 10,000	Multiple case series; no RCTs	Hemodynamically stable patients, low-to-moderate β-hCG	Breastfeeding, liver/renal disease, blood dyscrasias, immunodeficiency
Methotrexate (local, intra-sac or intra-cervical)	Direct cytotoxic effect, often combined with KCl for feticide	Faster vascular regression, less systemic toxicity, more targeted effect	Technically demanding; requires advanced ultrasound/hysteroscopic skills; incomplete effect possible	Case reports, small series	Viable, highly vascular CP/HCP in specialized centers	Same as systemic MTX; also contraindicated if technical expertise unavailable

Adapted from Gómez García et al., 2012 (Eur J Obstet Gynecol Reprod Biol) [6]; Stika 2012 (Clin Obstet Gynecol) [21]; Yamaguchi et al., 2017 (Ultrasound Obstet Gynecol) [22]; Petousis et al., 2015 (World J Clin Cases) [8]; Heikinheimo et al., 2004 (Acta Obstet Gynecol Scand) [24]; Kung & Chang 1999 (AJOG) [37].

**Table 2:** Published research on cervical and HCP.

Study (Year)	Design and N	Main Treatment(s)	Key Outcomes	Subsequent Fertility
Jeng et al., 2007 (Obstet Gynecol) [36]	Prospective series, 38	TVUS-guided intra-sac MTX (50 mg); + KCl if FHR present	Conservative ablation in early CP with uterine preservation; ultrasound-guided protocol standardized across centers.	Paper focuses on technique; uterus preserved; later conception potential discussed but not quantified.
Fylstra, 2014 (AJOG) [45]	Retrospective series, 13	Suction curettage + balloon tamponade	13/13 successful first-trimester terminations with hemostasis using immediate balloon tamponade; outpatient-style technique.	Not the primary endpoint; fertility not systematically reported.
Hu et al., 2016 (BJOG) [46]	Retrospective series, 19	UAE → curettage (24–72 h)	0 hysterectomies; safe and effective hemostasis.	Among 9 with follow-up (median 59 mo), 8 resumed normal menses; 1 term birth.
Hirakawa et al., 2009 (AJR) [47]	Retrospective series, 8	UAE + MTX	Effective bleeding control with uterine preservation; no UAE-related complications reported.	Fertility preserved in follow-up (details limited).
Zakaria et al., 2011 [51]	Retrospective series, 15	MTX ± Leucovorin alone (n = 5) vs. +UAE (n = 6) vs. +UAE + KCl (n = 4)	No UAE complications; stratified by markedly higher initial β-hCG in UAE groups.	Of 10 who had UAE, 2 later had viable pregnancies.
Kim et al., 2004 (J Korean Med Sci) [35]	Retrospective series, 31	Systemic MTX vs. non-MTX conservative	Uterus preserved in all; authors emphasize conservative success and bleeding avoidance.	3 patients achieved subsequent live births; paper also notes 2 heterotopic CP cases managed conservatively.
Kung & Chang, 1999 (AJOG) [37]	Retrospective series, 62	MTX-based (viable vs. non-viable stratified)	High overall conservative success; viability influenced need for adjuncts.	Authors report preserved reproductive capacity (details in full text).
Uludag et al., 2017 (J Obstet Gynaecol Res) [52]	Single-center series, 10	Systemic or local MTX	Conservative treatment effective in early CP with high β-hCG; protocolized MTX use.	Fertility outcomes not fully detailed in abstract.
Mori et al., 2022 (RBGO) [53]	Tertiary-service series, 13	Mostly MTX ± procedure (curettage/Foley)	12/13 managed conservatively; 1 hysterectomy for instability.	Not systematically reported.
Tanos et al., 2019 (J Gynecol Obstet Hum Reprod) [38]	Case series, 4	Hysteroscopic resection (often with MTX)	All resolved with minimal blood loss and short stays; illustrates endoscopic option.	Not evaluated.
Kochi et al., 2014 (J Obstet Gynaecol Res) [54]	Case series, 4	Intra-arterial MTX via uterine arteries; +UAE if heavy bleeding	Rapid resolution (≤8 days), uterine preservation across cases.	Not evaluated.
Krissi et al., 2014 (Eur J Obstet Gynecol Reprod Biol) [55]	Retrospective series, 25 (includes 10 CP)	Uterine artery MTX infusion + UAE + systemic MTX	96% overall success; mild transient side effects; offers real-world safety data for UAE-based protocols.	Paper reports future fertility experience within cohort (mixed NT-EP).

Selected published manuscripts on CP reporting conservative pharmacologic and/or minimally invasive surgical management, hemostatic efficacy, and fertility outcomes, where available. Studies are ordered by breadth/impact and recency. Abbreviations: MTX = methotrexate; KCl = potassium chloride (feticide); TVUS = transvaginal ultrasound; FHR = fetal heart rate; UAE = uterine artery embolization. “Conservative” indicates uterus-preserving therapy (medical and/or minimally invasive surgical). Some mixed non-tubal ectopic series are included when CP-specific data are extractable.

## Data Availability

No new data were created or analyzed in this study.

## Abbreviations

The following abbreviations were used in this manuscript:

anti-D	anti-D immunoglobulin (Rh[D] immune globulin)
D&C	dilation and curettage
FHR	fetal heart rate
HCP	HCP
IM	intramuscular
IUP	intrauterine pregnancy
IV	intravenous
KCl	potassium chloride
MRI	magnetic resonance imaging
MTX	methotrexate
PO	per os (oral)
q6h	every 6 h
q8h	every 8 h
TVUS	transvaginal ultrasound
UAE	uterine artery embolization
β-hCG	beta-human chorionic gonadotropin
